# Long noncoding RNAs are involved in multiple immunological pathways in response to vaccination

**DOI:** 10.1073/pnas.1822046116

**Published:** 2019-08-09

**Authors:** Diógenes S. de Lima, Lucas E. Cardozo, Vinicius Maracaja-Coutinho, Andreas Suhrbier, Karim Mane, David Jeffries, Eduardo L. V. Silveira, Paulo P. Amaral, Rino Rappuoli, Thushan I. de Silva, Helder I. Nakaya

**Affiliations:** ^a^Department of Clinical and Toxicological Analyses, School of Pharmaceutical Sciences, University of São Paulo, 05508-000 São Paulo, Brazil;; ^b^Advanced Center for Chronic Diseases (ACCDiS), Facultad de Ciencias Químicas y Farmacéuticas, Universidad de Chile, 8380492 Santiago, Chile;; ^c^Inflammation Biology Laboratory, QIMR Berghofer Medical Research Institute, Brisbane, QLD 4029, Australia;; ^d^Vaccines and Immunity Theme, Medical Research Council Unit, The Gambia at LSHTM, Banjul, The Gambia;; ^e^The Gurdon Institute, University of Cambridge, CB2 1QN Cambridge, United Kingdom;; ^f^GlaxoSmithKline, 53100 Siena, Italy;; ^g^Department of Infectious Diseases, Imperial College London, W12 ONN London, United Kingdom;; ^h^Centre of International Child Health, Section of Paediatrics, Department of Medicine, Imperial College London, W2 1PG London, United Kingdom;; ^i^Department of Infection, Immunity & Cardiovascular Disease, University of Sheffield, S10 2RX Sheffield, United Kingdom;; ^j^Scientific Platform Pasteur, University of São Paulo, 05508-210 São Paulo, Brazil

**Keywords:** systems biology, vaccination, transcriptome, long noncoding RNAs

## Abstract

Understanding the mechanisms of vaccine-elicited protection contributes to the development of new vaccines. The emerging field of systems vaccinology provides detailed information on host responses to vaccination and has been successfully applied to study the molecular mechanisms of several vaccines. Long noncoding RNAs (lncRNAs) are crucially involved in multiple biological processes, but their role in vaccine-induced immunity has not been explored. We performed an analysis of over 2,000 blood transcriptome samples from 17 vaccine cohorts to identify lncRNAs potentially involved with antibody responses to influenza and yellow fever vaccines. We have created an online database where all results from this analysis can be accessed easily. We found that lncRNAs participate in distinct immunological pathways related to vaccine-elicited responses. Among them, we showed that the expression of lncRNA FAM30A was high in B cells and correlates with the expression of immunoglobulin genes located in its genomic vicinity. We also identified altered expression of these lncRNAs in RNA-sequencing (RNA-seq) data from a cohort of children following immunization with intranasal live attenuated influenza vaccine, suggesting a common role across several diverse vaccines. Taken together, these findings provide evidence that lncRNAs have a significant impact on immune responses induced by vaccination.

## Significance

Long noncoding RNAs (lncRNAs) are known to be involved in several immunological processes. In spite of their general relevance to human immunity, to date there are no reports on the importance of lncRNAs in vaccine responses. Here we apply a “systems vaccinology” framework to study the role of lncRNAs in vaccine-mediated immunity. We applied meta-analytical approaches using public microarray data from over 2,000 blood transcriptome samples of vaccinees and an RNA-sequencing (RNA-seq) dataset from vaccinated children to tackle this question. Our results indicate that lncRNAs are important players in several immunological processes elicited by vaccination.

Vaccines are among the greatest achievements of medicine, having greatly reduced the mortality and morbidity of many major infectious diseases of mankind (1). However, our understanding of the molecular mechanisms involved in vaccine-mediated protection remains incomplete (2). The emerging field of systems vaccinology provides substantial new insights and a new tool for testing and developing vaccines (2). This approach has been applied to the study of immune responses induced by yellow fever vaccination (3–5), influenza vaccination (6–13), malaria (14), and herpes zoster vaccination (15) among others.

The human genome may transcribe over 100,000 long noncoding RNAs (lncRNAs), which are defined as transcripts with more than 200 nucleotides without detectable coding potential (16). While initially considered transcriptional noise, lncRNAs are increasingly linked to multiple biological processes ranging from chromatin remodeling (17) to the regulation of splicing patterns of transcripts (18). Moreover, several lncRNAs are known to exert functions in immune-related cells and processes, such as control of dendritic cell differentiation (19), cytokine production (20), activation of CD8+ T cells (21), control of Foxp3 expression in regulatory T cells (22), and CD4+ T cell differentiation (23).

Systems vaccinology studies have focused solely on analyzing the expression of genes that encode proteins. Only one of these studies proposed that noncoding transcripts may play a role in vaccine-induced immunity (7). Given the compelling body of knowledge associating lncRNAs with immunological processes, we hypothesized that they might also play a key role in vaccine-induced immunity. Leveraging the fact that most microarray platforms contain probes targeting lncRNAs (24, 25), we performed a massive transcriptome analysis of 2,059 blood samples from 17 cohorts of participants vaccinated with yellow fever or influenza vaccine. Our analysis identified hundreds of lncRNAs potentially involved with the immune responses elicited by these vaccines. Specifically, we show herein that lncRNAs present reiterated changes in expression following vaccination and that these changes are correlated to antibody production. We also propose that vaccine-related lncRNAs may exert their activities in distinct immunological pathways. Moreover, we used an RNA-sequencing (RNA-seq) dataset to identify lncRNAs which were induced or repressed after vaccination with a live attenuated influenza vaccine (LAIV). Finally, we created an online database that stores all of the results from our meta-analysis. Taken together, our findings reveal the potential roles of lncRNAs in regulating the immune responses to vaccination.

## Results

### Vaccine Cohorts and Reannotation of Microarray Platforms.

**Fig. 1.**
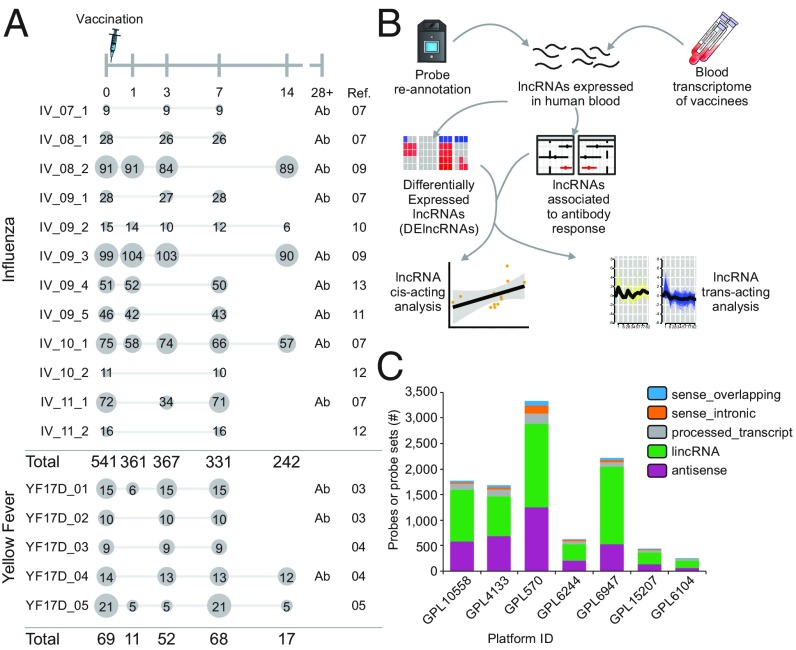
Experimental design of meta-analysis of 17 vaccine cohorts. (*A*) Graphic representation of sampling regimen. Blood samples were collected on days 0, 1, 3, 7, 14, and 28 after vaccination with IV or YF-17D. Numbers in circles represent the number of samples used from each cohort after processing. The Influenza season is shown in the name of the IV cohort (07 = 2007/2008, 08 = 2008/2009, etc.). (*B*) Summary of computational analyses performed in this work. Blood transcriptome from cohorts was assessed with commercial microarray platforms. After remapping of probes, each cohort was subjected to analysis of differential expression, correlation to antibody titers, correlation to neighboring genes, and coexpression analysis. (*C*) Characterization of lncRNA classes represented in each microarray platform.

Blood and peripheral blood mononuclear cell (PBMC) expression data from 17 cohorts of human participants immunized with either yellow fever vaccine (YF-17D) or inactivated influenza vaccine (IV) were analyzed (Fig. 1*A* and *SI Appendix*, Table S1). Most of these cohorts included antibody titer data collected before and after vaccination (Fig. 1*A*). RNA derived from these samples was hybridized onto microarrays, and raw data were deposited in the Gene Expression Omnibus (GEO) and ArrayExpress databases. After data preprocessing, four types of analyses for each dataset were conducted to identify lncRNAs associated with vaccine-induced immune responses (Fig. 1*B* and *Methods*).

To identify probes representing lncRNAs, the probe sequences from each microarray platform were aligned to the human genome assembly hg38 and were reannotated based on genomic features described in GENCODE version 24. All microarray platforms contained probes targeting different classes of lncRNAs (Fig. 1*C* and *SI Appendix*, Fig. S1 *A* and *B*). Some probes that were officially annotated by the microarray manufacturer as representing protein-coding genes were, in reality, targeting antisense, intronic, or intergenic lncRNAs (*SI Appendix*, Fig. S2). Since each microarray platform contains probes targeting specific sets of genes and lncRNAs, we sought to characterize intersections between platforms. A total of 204 and 91 lncRNAs were detected simultaneously in all microarray platforms used in IV and YF-17D vaccine cohorts, respectively. These numbers increased when different combinations of microarray platforms were considered (*SI Appendix*, Fig. S1 *A* and *B*).

### lncRNAs Present Coherent Changes of Expression after Vaccination.

**Fig. 2.**
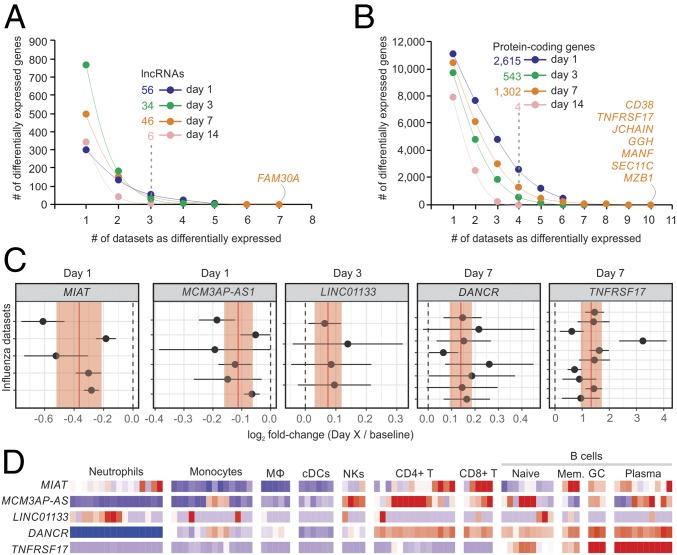
Transcriptome analysis of cohorts immunized with inactivated IV. Cumulative sum of differentially expressed lncRNAs (*A*) and protein-coding genes (*B*) (*y*-axis) in one or more datasets (*x*-axis). Transcripts were considered differentially expressed if limma *P* values were lower than 0.05 in at least three cohorts (for lncRNAs) or four cohorts (for protein-coding genes). (*C*) Forest plots of representative lncRNAs and *TNFRSF17* with reiterated differential expression. Log_2_ fold changes with their corresponding 95% confidence interval (*x*-axis) are plotted for each cohort (*y*-axis). Red vertical lines and shaded regions represent log_2_ fold-change summaries and their 95% confidence intervals, respectively. (*D*) Heatmap depicting gene expression of human immune cells from ref. 28. Logs (FPKM) of genes are scaled around zero. Columns represent samples; rows represent genes.

Dozens of lncRNAs were identified whose expression was induced or repressed after 1, 3, 7, or 14 d following IV compared with baseline (Fig. 2*A* and *SI Appendix*, Fig. S3). Among the protein-coding genes, TNFRSF17, GGH, and CD38 were up-regulated 7 d postvaccination in most cohorts (Fig. 2 *B* and *C*), as previously reported (6). Gene set enrichment analysis (GSEA) was performed for each comparison using blood transcription modules (BTMs) (26). For IV, signatures related to monocytes, cell cycle, TLR signaling, antigen presentation, and IFN response were mostly induced on day 1 postvaccination, whereas a strong signature associated with B cells and stimulated CD4+ T cells were detected in the majority of cohorts on day 7 postvaccination (*SI Appendix*, Fig. S4).

We also performed random-effects meta-analyses (27) to identify lncRNAs with coherent differential expression throughout the IV and YF-17D cohorts. Often, lncRNAs showed subtle but significant changes in expression following vaccination. For the IV cohorts, MIAT and MCM3AP-AS1 were down-regulated on day 1 postvaccination (Fig. 2*C*) (nominal *P* values = 3.05 × 10^−6^ and 1.06 × 10^−5^, respectively). LINC01133 and DANCR were up-regulated on day 3 (nominal *P* value = 0.000917) and on day 7 (nominal *P* value = 1.05 × 10^−8^) postvaccination, respectively (Fig. 2*C*). As expected, the protein-coding gene TNFRSF17 was up-regulated on day 7 post-IV in all cohorts (nominal *P* value = 1.787 × 10^−11^) (Fig. 2*C*). For YF-17D–vaccinated cohorts, differentially expressed lncRNAs included AATBC (nominal *P* value = 0.0008938 on day 7), MAPKAPK5-AS1 (nominal *P* value = 0.0005284 on day 7), and LRRC75A-AS1 (nominal *P* value = 0.000339 on day 3 and 2.524 × 10^−8^ on day 7). Increases in the expression of IFN-stimulated genes after vaccination is a mark of YF-17D response (3, 4). Accordingly, we detected sharp and coherent increases in the expression of IFI27 in all YF-17D cohorts on day 7 (nominal *P* value = 6.393 × 10^−45^) (*SI Appendix*, Fig. S5).

Next we investigated the expression of these lncRNAs in different immune-related cells using RNA-Seq data from Blueprint (28) (see *Methods*, Fig. 2*D*, and *SI Appendix*, Fig. S4). DANCR, which had previously been implicated in the control of histone methylation mediated by EZH2 (29, 30), is specifically expressed in cells of lymphoid origin (Fig. 2*D*). The lncRNA AATBC, which is associated with cell proliferation and apoptosis (31), was expressed specifically in monocytes (*SI Appendix*, Fig. S5).

### FAM30A lncRNA Levels Correlate with Those from Neighboring Immunoglobulin Heavy Genes.

**Fig. 3.**
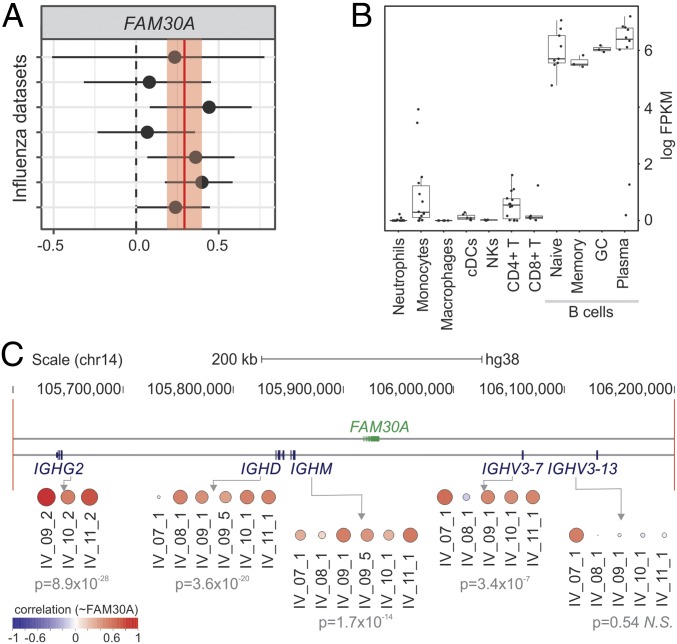
*FAM30A* expression correlates to antibody production and to gene segments within the IgH locus. (*A*) Correlations between *FAM30A* and antibody titers with corresponding 95% confidence interval (*x*-axis) are plotted for each cohort (*y*-axis). Red vertical line and shaded region represent the correlation summary and its 95% confidence interval, respectively. (*B*) *FAM30A* expression is higher in B cells according to ref. 28. (*C*) Fold-change correlation between FAM30A and gene segments within the IgH locus at day 7 after inactivated IV. Each circle represents Pearson correlation coefficient in different IV cohorts. Meta-analysis *P* values are represented below cohort identifiers.

We also identified lncRNAs whose expression correlated with antibody response in both IV and YF-17D cohorts. The lncRNA with the highest correlation throughout all IV cohorts was FAM30A (KIAA0125), a lncRNA embedded in the immunoglobulin (Ig) heavy (IgH) locus on chromosome 14, in antisense orientation to IgH gene segments (Fig. 3). The expression of FAM30A showed a positive correlation to the levels of antibody titers on day 7 postvaccination in most of the IV cohorts (Fig. 3*A*). Higher expression levels of FAM30A were seen in B cells compared with other immune cell types (Fig. 3*B*). Increases in the frequency of antibody-secreting cells (ASCs) could be observed 7 d post-IV (11, 13) and may partly explain the observed positive correlation between FAM30A expression and antibody titers seen in the microarray analysis. To check for evidence of coregulation, we compared the expression levels of FAM30A with those of 4 IgH locus genes located in its genomic vicinity on day 7 post-IV. This approach revealed a strong positive correlation with IgH gene segments (Fig. 3*C*), suggesting potential related functions and a *cis*-regulatory role for FAM30A in regulating the expression and functional organization of the gene segments. We describe examples of lncRNAs whose expression is inversely correlated with antibody responses in the *SI Appendix*.

### Modular Expression Analyses of lncRNAs.

**Fig. 4.**
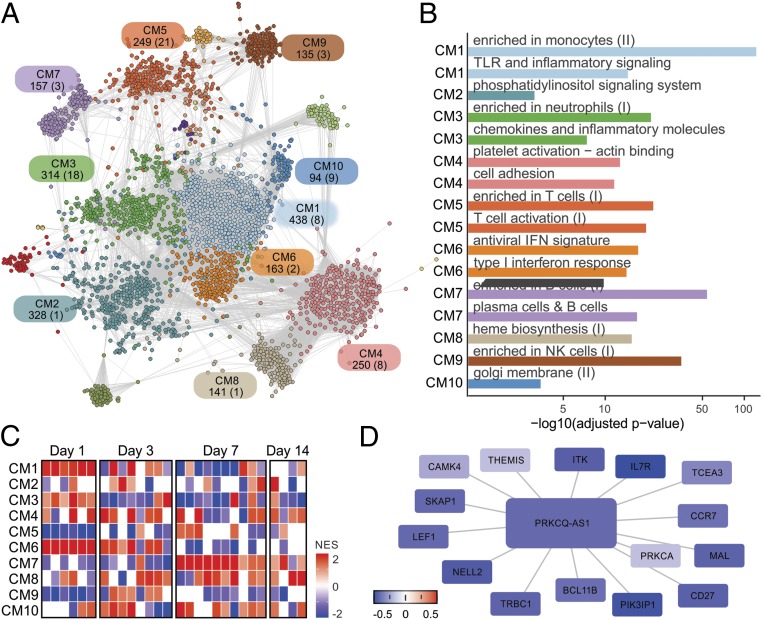
Influenza vaccination consensus network. (*A*) The consensus network was constructed by intersecting CEMiTool results from all IV cohorts and prioritizing frequently detected edges. Inference of communities was performed using a spin glass clustering algorithm. Graph colors are based on community assignment. Each community is represented by a rectangle containing its name and the number of genes and lncRNAs (in parentheses). (*B*) Overrepresentation analysis of selected communities using blood transcriptional modules (BTMs). False discovery rates (FDR) (*x*-axis) are plotted for each BTM. (*C*) Gene set enrichment analysis (GSEA) performed with network communities (rows) and mean fold changes of all vaccinees from each cohort as ranks (columns). Heat map represents normalized enrichment scores (NESs) of communities whose FDR < 0.05. (*D*) Some PRKCQ-AS1 connections within CM5. The colors of nodes represent log_2_ fold-change summaries on day 1 relative to baseline.

Assessing gene coexpression patterns in blood transcriptome may help in the identification of novel functional connections between lncRNAs and mRNAs after vaccination (32). To detect shared coexpression patterns between IV cohorts, we first ran CEMiTool (33) in each cohort using samples from baseline to day 7 after vaccination. Next we integrated the resulting modules into a consensus network and used a spin glass method to identify 16 cohesive network subgroups (34), hereafter referred as communities (Fig. 4*A*). These communities were enriched for distinct immune-related cells and processes such as monocytes (CM1), platelet activation (CM4), T cells (CM5), IFN response (CM6), B cells (CM7), and natural killer cells (CM9) (Fig. 4*B*). GSEAs were then undertaken using the communities as gene sets and mean log_2_ baseline-normalized fold change from all IV cohorts as ranks. This approach revealed that communities displayed congruent expression patterns between different cohorts in equivalent comparisons. For example, genes from CM1, CM3, and CM6 were highly induced on day 1 postvaccination, whereas those from CM7 and CM8 were induced on day 7 postvaccination (Fig. 4*C*).

Network communities contained dozens of lncRNAs (*SI Appendix*, Table S2). Meticulous examination of genes connecting to these lncRNAs may unveil potential transregulatory relationships (see *SI Appendix*). For instance, lncRNA *PRKCQ-AS1* is a member of the T cell–related community CM5 in the IV network (Fig. 4*A*). GSEA revealed that CM5 is consistently down-regulated on day 1 after IV in all IV cohorts (Fig. 4*C*). A subnetwork of genes connected to PRKCQ-AS1 is also broadly down-regulated on day 1 (Fig. 4*D*). Several members of this subnetwork are well-known T cell–related genes, such as CCR7, CD27, ITK, LEF1, THEMIS, and SKAP1. These findings suggest that PRKCQ-AS1 may be involved with T cell functions.

The same modular analysis was performed with YF-17D cohorts, which resulted in 17 network communities (*SI Appendix*, Fig. S6). Several lncRNAs were found within communities related to “chemokines in myeloid cells,” “T cell differentiation,” and “platelet activation” (*SI Appendix*, Fig. S6). GSEA revealed that, while activation of IFN response was restricted to days 1 and 3 post-IV (CM6 in Fig. 4*C*), a strong activation of IFN response was still observed on day 7 in the YF network (CM2 in *SI Appendix*, Fig. S6*C*).

### Differential Expression of lncRNAs in RNA-Seq Data Generated from a New Pediatric Vaccine Cohort.

As RNA-seq analyzes the whole transcriptome, it has the ability to discover lncRNAs associated with vaccination. We assessed whether lncRNAs were differentially expressed in an RNA-seq dataset of 26 children aged 2–4 y old immunized with intranasal LAIV. Vaccination induced differential expression of 55 lncRNAs in whole blood at day 2 post-LAIV compared with baseline, with a false discovery rate (FDR) threshold of 0.05 (*SI Appendix*, Fig. S7). Several lncRNAs found in our meta-analyses were also found among differentially expressed genes in this cohort, including *FAM225A*, *LRRC75A-AS1*, *MAPKAPK5-AS1*, *DANCR*, and *DICER-AS1*.

### VaccineDB: A Systems Vaccinology Database.

We developed a user-friendly online database named VaccineDB (https://vaccinedb.sysbio.tools/) to catalog and organize the information generated in this work. By selecting the vaccine (IV or YF-17D), the type of analysis (differential expression, gene-antibody correlation, gene-lncRNA correlation), and gene symbol identifiers, the database displays one forest plot for each time point after vaccination (*SI Appendix*, Fig. S8). The differential expression and gene-antibody correlation analyses show, respectively, the expression changes and the antibody correlation values of user-input protein-coding genes or lncRNAs after either IV or YF-17D (*SI Appendix*, Fig. S8). The database also displays the correlation between the expression of a user-provided protein-coding gene and lncRNAs within the same genomic region. This approach may help identify lncRNAs with coordinated expression and potential regulatory *cis*-acting roles in response to vaccination. Finally, to verify if the lncRNA is associated with known SNPs, the database displays the genomic locus of the lncRNA and all SNPs from the dbSNP database located there.

## Discussion

Multiple orchestrated processes are involved in the immune response to vaccination and infection. Our analyses have revealed that lncRNAs may be linked to some of these processes. While YF-17D contains a live attenuated yellow fever virus that needs to replicate before priming the immune system of naïve individuals, IV contains proteins of the influenza virus that boost a preexisting immunity. At day 7 postvaccination, the blood signature of YF-17D vaccinees is related to innate antiviral IFN responses (3), whereas the signature of IV vaccines is more associated with antibody-secreting B cells (6, 7). These differences may explain the distinct set of lncRNAs associated with each vaccine. Nevertheless, we have still identified some commonly induced lncRNAs which may reflect underlying common pathways in successful generation of vaccine-induced immunity.

LncRNA transcripts whose functions have been described in other contexts, such as *MIAT*, *LINC01133*, and *DANCR*, were found to be consistently differentially expressed in multiple vaccination cohorts. *MIAT*, also known as Gomafu, was first described in 2006 as a transcript harboring SNPs conferring increased susceptibility to myocardial infarction (35). More recent reports have shown that *MIAT* might be involved in processes related to apoptosis, proliferation, oncogenesis, and cellular migration by several mechanisms (reviewed in ref. 36). Consistent down-regulation 1 d after IV across multiple cohorts may point to an unappreciated role for MIAT in the early immune response to vaccines. *LINC01133* expression was found to be increased in several cancers (37–39). In our analysis, LINC01133 was found to be up-regulated at day 3 after IV, which suggests that it may also act to regulate immune cell proliferation following vaccination. A role for lncRNAs in adaptive immune responses also needs consideration. For example, *DANCR*, which was also up-regulated in young vaccine responders at day 7, as discussed in a previous publication (12), was found to influence the activity of Enhancer of Zeste Homolog 2 (EZH2) in multiple cancer types (29, 30, 40). Epigenetic modifications mediated by EZH2 have been shown to affect B cell differentiation and antibody production in mice (41). *DANCR* up-regulation 7 d after IV suggests that it takes part in regulating the differentiation of antibody-secreting cells, whose frequency in peripheral blood rises at roughly 7 d after IV (11, 13).

Other lncRNAs, such as *MCM3AP-AS1*, *MAPKAPK5-AS1*, and *LRRC75A-AS1*, are still understudied. Nevertheless, their genomic position might give clues to their potential target genes. For example, *MCM3AP* encodes germinal center–associated nuclear protein (GANP), which is related to antigen-specific B cell proliferation in germinal centers (42). Down-regulation of *MCM3AP-AS1* 1 d after IV may point toward a role for this transcript in B cell activity. *MAPKAPK5* encodes a serine/threonine kinase that activates heat shock protein 27, which has been associated with replication of viruses (43). Down-regulation of *MAPKAPK5-AS1* 7 d after YF-17D and 2 d after LAIV suggests a potential role for this transcript in replication of attenuated viral vaccines. Furthermore, down-regulation of *LRRC75A-AS1* at days 1 and 7 after YF-17D (*SI Appendix*, Fig. S4), day 2 after LAIV (*SI Appendix*, Fig. S7), and day 1 after IV (*SI Appendix*, Fig. S8) suggests a common role for this lncRNA in response to more than one vaccine.

By correlating changes in gene expression with fold increases in antibody titers following IV, we identified *FAM30A*, a transcript in antisense orientation in relation to gene segments from the IgH locus. *FAM30A* was first described as a cDNA expressed in the KG-1 cell line, spleen, and peripheral blood (44). Antisense noncoding expression within murine loci poised for recombination has been reported in mice (45–47). Although conclusive functional characterizations of these mouse lncRNAs are still lacking, they were proposed to act in the regulation of variable (diversity) joining [V(D)J] recombination by mediating long-range DNA interactions between Ig segments (46). Network analysis also revealed that *FAM30A* is a member of CM7, a community associated with B cell–related genes (Fig. 4*B* and *SI Appendix*, Table S2). Altogether, our results indicate that *FAM30A* is probably related to the biology of B lymphocytes and to antibody responses elicited by IV. Although *FAM30A* is not conserved in mice, therefore preventing murine studies, its association with immune responses to vaccination warrants further investigation. Because microRNAs (48–50) and linc-MAF-4 (51), a long intergenic noncoding RNA, can be used as biomarkers of lymphocyte activation and differentiation, we propose that *FAM30A* could be useful to quickly monitor the antibody responses induced by influenza vaccination.

The limitations of this study must be mentioned. Since probes from different manufacturers may be designed to target different regions of the same gene, heterogeneity in expression between cohorts might occur due to differential exon usage. Moreover, the occurrence of correlations between pairs of neighboring genes is not definitive evidence for the existence of regulatory relationships (52). Our findings thus suggest the existence of regulatory functions, but are not definitive evidence for such activities. Further functional validation experiments are required (53).

To the best of our knowledge, there is no study focusing on the role of lncRNAs during vaccine-induced immunity. The work presented herein implicates lncRNAs in the regulation of specific processes associated with vaccination. We have also created an online database where users can submit personalized queries for coding genes and lncRNAs to visualize the results from our meta-analyses. We hope that this comprehensive resource will aid researchers in rapidly assessing hypotheses related to the blood transcriptomics of human vaccines.

## Methods

See *SI Appendix*, *Supplementary Materials and Methods*, for details and additional references.

### Vaccination of Human Subjects and Data Collection.

Datasets with participants immunized with inactivated IVs or with YF-17D were used (*SI Appendix*, Table S1). PBMCs or whole blood microarray data from young adult subjects (20–49 y), which were collected before and after vaccination, were downloaded from GEO (https://www.ncbi.nlm.nih.gov/geo/) and ArrayExpress (https://www.ebi.ac.uk/arrayexpress/).

### Microarray Platform Reannotation Pipeline.

We developed a Snakemake pipeline to update probe annotations from microarray platforms. Probe sequences were aligned against the HG38 assembly of the human genome using BLAT (54) with thresholds of alignment scores > 90% and identity scores > 90%. Probes with multiple alignments were discarded, and remaining probes were reassigned to gene feature annotations provided by Gencode version 24 using BEDTools (55). Probes aligning with more than one feature were also discarded.

### Vaccination with Live Attenuation Influenza Vaccine and RNA Sequencing.

Children aged 2–4 y old (*n* = 52) were immunized with Russian-backbone intranasal LAIV (Serum Institute of India Pvt Ltd) and RNA extracted from whole blood collected in PAXgene tubes taken at baseline and at day 2 after vaccination (clinicaltrials.gov identifier NCT02972957). This study was approved by The Gambia Government and UK Medical Research Council joint ethics committee and the Medicines Control Agency of The Gambia, and it was done according to International Conference on Harmonisation Good Clinical Practice standards. A parent provided written or thumbprinted informed consent for their child or children to participate. If parents were not English-literate, an impartial witness was present throughout the informed consent discussion undertaken in a local language, who signed to confirm completeness of the consent provided. Library preparation and RNA sequencing (Illumina Hiseq X; 100-bp paired-end reads) were performed at the Beijing Genome Institute. Quality control of raw sequencing data was performed using FastQC. Mapping to the human reference genome (GRCh38) was done using DART (56) (version 1.3.0). For each sample, histograms of distance (within tile) between wells of all duplicate alignments were evaluated. A distance of 2,500 pixels was appropriate to filter out likely exclusion amplification duplicates. Picard MarkDuplicates was used to remove these duplicates. Reads were counted at gene level using featureCounts (57) via the wrapper R package Rsubread. Raw and processed data are available in the GEO database (GSE128224).

### Differential Expression.

Differential expression analysis was conducted using a moderated *t* test provided by the limma package (58). Comparisons were performed between expression values from days 1, 3, 7, and 14 after vaccination and day 0 (baseline). Analyses were paired by subject and performed separately for each cohort. The edgeR package (59) was used in the differential expression analysis conducted with RNA-seq data from children vaccinated with LAIV.

### Correlation with Immune Parameters.

Antibody titers, assessed with either hemagglutination-inhibition assay (HAI) or microneutralization assay (MN), were retrieved from microarray databases or supplemental materials from articles. Antibody titer fold increases from each subject between day 28, 63, or 70 after vaccination and baseline values were log-transformed. Pairwise log_2_ fold-change values for each day after vaccination (compared with baseline) were correlated (Pearson’s correlation) with corresponding antibody titer fold increases.

### Correlation of Coding and Neighboring Noncoding Transcripts.

A catalog of lncRNA-mRNA genomic neighboring pairs was assembled using the GenomicRanges package (60). Pairwise log_2_ fold-change values for each day after vaccination compared with baseline of neighboring pairs were correlated (Pearson correlation) to infer potential *cis*-regulatory regions.

### Random-Effects Meta-Analyses.

Effect sizes from differential expression analysis, neighboring gene pair correlation analysis, and antibody titer correlation analysis were integrated using random-effects models for meta-analyses, as implemented in the metafor package (27). Limma log_2_ fold changes from each study and their corresponding variances were submitted to the rma function for estimation of summarized effect sizes using restricted maximum likelihood (REML). For correlation-based analyses, sampling variances of Pearson correlation coefficients (PCCs) from each study were estimated using the escalc function. Then PCCs and variances were submitted to the rma function for estimation of summarized effect sizes using Hunter and Schmidt (HS). Studies were weighted by the inverse of variance.

### Building of a Consensus Network.

Samples collected between days 0 and 7 after IV were submitted to CEMiTool using default parameters (Pearson correlation coefficient and unbiased selection of genes by a variance-based filter) (33). Genes from each module were connected between themselves to create fully connected subnetworks for each cohort. The Pearson correlation coefficient between each pair of genes (edge) in each cohort was then computed.

## Supplementary Material

Supplementary File
