# Supplementary material for: Long noncoding RNAs are involved in multiple immunological pathways in response to vaccination
Source: Proc Natl Acad Sci U S A. 2019 Aug 9;116(34):17121–6. doi: 10.1073/pnas.1822046116 (PMC6708379; doi:10.1073/pnas.1822046116)
Supplement: Supplementary File [file pnas.1822046116.sapp.pdf]

Supplementary Information

Long non-coding RNAs are involved in multiple immunological pathways in response to vaccination

Authors and affiliations

Diógenes S. de Lima[1], Lucas E. Cardozo[1], Vinícius Maracaja-Coutinho[2], Andreas Suhrbier[3], Karim Mane[4], David Jeffries[4], Eduardo L. V. Silveira[1], Paulo P. Amaral[5], Rino Rappuoli[6*], Thushan I. de Silva[4,7], Helder I. Nakaya[1*]

[1] Department of Clinical and Toxicological Analyses, School of Pharmaceutical Sciences, University of Sao Paulo, SP (05508-000), Brazil

[2] Advanced Center for Chronic Diseases - ACCDiS, Facultad de Ciencias Químicas y Farmacéuticas, Universidad de Chile, Santiago, Chile.

[3] Inflammation Biology Laboratory, QIMR Berghofer Medical Research Institute, Brisbane, Qld. 4029, Australia

[4] Vaccines and Immunity Theme, Medical Research Council Unit The Gambia at LSHTM, PO Box 273, Banjul, The Gambia.

[5] The Gurdon Institute, University of Cambridge, Tennis Court Road, Cambridge, CB2 1QN, United Kingdom.

[6] Chief Scientist & Head of External Research and Development. GSK , via Fiorentina 1, 53100 Siena , Italy

[7] Centre of International Child Health, Section of Paediatrics, Department of Medicine, Imperial College London, St Mary's Campus, London W2 1PG, UK.

www.pnas.org/cgi/doi/10.1073/pnas.1822046116

SI Methods

Quality control and preprocessing of microarray samples

Raw data from microarray datasets were submitted to arrayQualityMetrics package for aberrant sample detection (1). Samples flagged as outliers by two of the three tests performed by the package were removed. The resulting set of samples were iteratively resubmitted to arrayQualityMetrics using the same criteria until no samples were removed. Microarray datasets were then log transformed and quantile normalized. Samples from illumina platforms were normalized using functions provided by limma package (2), whereas samples from Affymetrix platforms were normalized using Robust Multi-Array Average (RMA) from affy (3). Probes targeting genes in common were summarized by selecting the ones showing the highest mean of expression between all samples. Genes whose mean of expression were in the first decile were removed.

Microarray platform reannotation pipeline

We developed a *Snakemake* pipeline to update probe annotations from microarray platforms. Probe sequences were aligned against the HG38 assembly of the human genome using BLAT (4) with thresholds of alignment score > 90% and identity score > 90%. Probes with multiple alignments were discarded and remaining probes were reassigned to gene feature annotations provided by Gencode v24 using Bedtools (5). Probes aligning with more than one feature were also discarded.

Correlation with immune parameters

Antibody titers, assessed either with Hemagglutination-inhibition assay (HAI) or Microneutralization assay (MN), were retrieved from microarray databases or supplemental materials from articles. Antibody titer fold increases from each subject between day 28, 63 or 70 after vaccination and baseline values were log transformed. Pairwise $\log_2$ fold change values for each day after vaccination (compared to baseline) were correlated (Pearson's correlation) with corresponding antibody titer fold increases.

Correlation between coding and neighboring non-coding transcripts

A catalog of lncRNA-mRNA genomic neighboring pairs was assembled using GenomicRanges package (6). Pairwise log_2 fold-change values for each day after vaccination compared to baseline of neighboring pairs were correlated (Pearson's correlation) to infer potential cis-regulatory regions.

Building of a consensus network

Samples collected between days 0 and 7 after IV were submitted to CEMiTool using default parameters (Pearson's correlation coefficient and unbiased selection of genes by a variance-based filter) (7). Genes from each module were connected between themselves to create fully connected subnetworks for each cohort. We then computed the Pearson's correlation coefficient between each pair of genes (edge) in each cohort. The resulting networks were then pruned by selecting edges that: 1) Were represented in at least two cohorts; 2) Had a mean correlation coefficient of greater than 0.6 if edge was represented in two cohorts; 3) Had a mean correlation coefficient of greater than 0.55 if edge was represented in three cohorts; 4) Had a mean correlation coefficient of greater than 0.5 if edge was represented in four cohorts; 5) Had a mean correlation coefficient greater of than 0.45 if edge was represented in five cohorts (only IV); 6) Were represented in 6 or more cohorts for IV and 5 or more cohorts for YF17D. Communities were inferred from resulting consensus network with a spin glass method implemented in igraph R package (https://igraph.org/r/).

Enrichment analysis and Over-Representation Analysis.

Gene Set Enrichment Analysis (GSEA) was performed in pre-ranked lists of individual log_2 fold-change values using fgsea package (Bioconductor version: Release 3.8). Over-Representation Analyses were performed using clusterProfiler R package (8). P-values were adjusted, and pathways with False Discovery Rates < 0.05 were selected. Blood Transcriptional Modules (BTMs) defined in (9) were used in both analyses.

Immune cell gene expression

Gene expression data from Blueprint (10) was retrieved using Deep Blue Epigenomic Data Server Application Programming Interface (API) (11). Queries for 'B-cell', 'T-cell', 'monocyte', 'blood', 'dendritic cell', 'macrophage', 'eosinophil', 'basophil',

‘neutrophil’, 'natural killer cell’ and 'hematopoietic stem cell' were submitted to the API. Fragments Per Kilobase Million (FPKM) expression data of genes defined in Gencode v24 for cell types matching queries were log transformed.

IV and YF17D vaccination consensus networks

We compared the YF17D and IV networks and found 86 connections between lncRNAs and protein-coding genes that were shared to both vaccines. These connections included FAM30A and the host gene of miR-22 among others. Most connections between lncRNAs and mRNAs were unique to IV network (2,177) or YF17D network (233), suggesting that different lncRNA-mediated regulation may be involved.

Examples of lncRNAs whose expression is inversely correlated with antibody responses post IV

1. GHRLOS

This lncRNA is antisense to ghrelin/obestatin prepropeptide gene (*GHRL*). The expression of *GHRLOS* at day 1 post-vaccination is inversely correlated with antibody responses (at day 30) in three IV cohorts. The expression of the sense gene *GHRL* at day 1 is also inversely correlated with antibody responses (at day 30) in three IV cohorts.

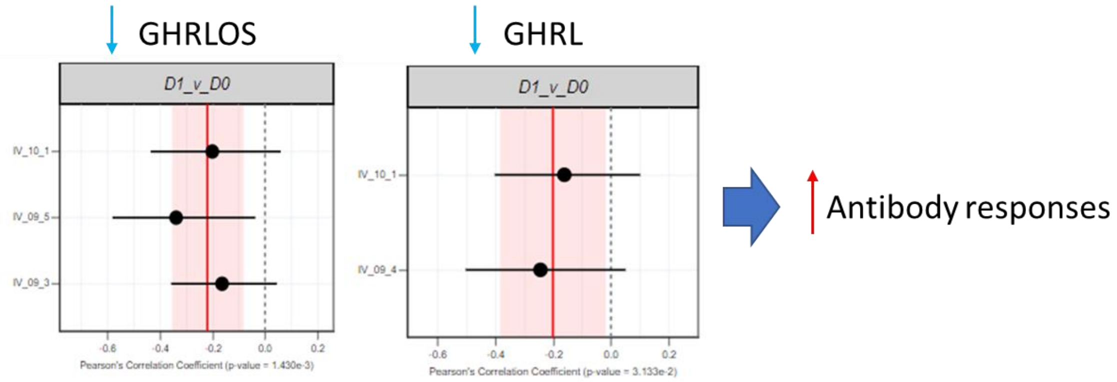

The sense gene *Ghrelin* plays a critical role in energy homeostasis and it is known as the "hunger hormone". Vishwa et al. (12) showed that ghrelin is expressed in human T cells and it may inhibit proinflammatory cytokines (e.g. TNF, IL-6, IL1B, among others). We suggest that regulation of GHRLOS/GHRL may contribute with the antibody responses induced by IV.

2. TRIM52-AS1

The expression of lncRNA TRIM52-AS1 at day 3 post-IV is inversely correlated with antibody responses (at day 30) in five IV cohorts (p = 0.00034). Although the TRIM52-AS1 is antisense to TRIM52, the expression of the gene TRIM52 is not correlated with antibody response.

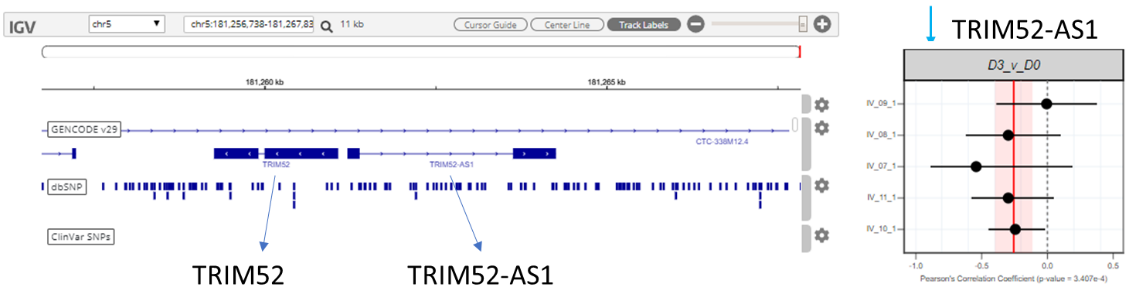

One study shows that down-regulation of TRIM52-AS1 is associated with cell proliferation (13). The Expression Atlas portal (14) shows that TRIM52-AS1 is also down-regulated in Th1 and Th2 polarised T cells (see below). This suggests that the lncRNA may be involved with CD4 T helper differentiation and proliferation.

Adj. P	Log_2 fold-change	Log_2-fold change	Species	Gene name	Comparison	Experimental variables	Experiment name
0.0003	- 3.7			TRIM52-AS1	'T-helper 1 polarising' at '5 days' vs 'no stimulus'	cell type, sampling time point, stimulus	Transcription profiling by array of human CD4+T-cells, Th1/Th2 polarized time-series and primary memory subsets
0.0003	- 3.3			TRIM52-AS1	'T-helper 2 polarising' at '5 days' vs 'no stimulus'	cell type, sampling time point, stimulus	Transcription profiling by array of human CD4+T-cells, Th1/Th2 polarized time-series and primary memory subsets

3. C1orf132 (MIR29C Host Gene)

The current annotation of lncRNA C1orf132 is MIR29B2CHG which is a host gene of two miRNAs: MIR29C and MIR29B2. The expression of lncRNA C1orf132 at day 7 post-IV is inversely correlated with antibody responses (at day 30) in six IV cohorts (p = 0.0017).

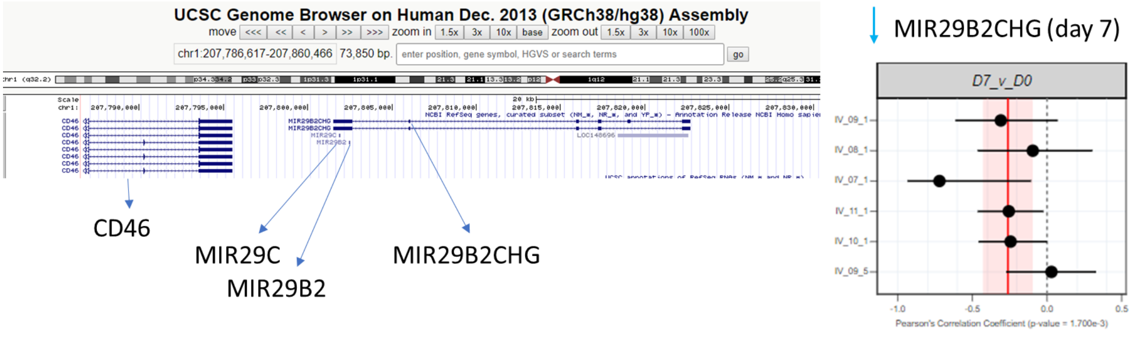

Several studies have shown that the miR-29 family may play a key role in adaptive immunity (15). Chun-Mei et al have shown that miR-29c may suppress the Tumor necrosis factor alpha-induced protein 3 (TNFAIP3), which is a key regulator in inflammation and immunity (16). Since TNFAIP3 is required for the normal

differentiation of the marginal zone B and B1 cell subsets in mice (17), we hypothesize that lower levels of miR-29c may be associated with higher antibody-secreting cells by increasing the TNFAIP3 protein levels.

Insights from Influenza vaccination consensus network (Figure 4)

The different cell populations activated upon vaccination may impact the functional status of subsets. For example, the community containing genes associated with antibody-secreting cells (ASCs) is CM7 (Figure 4a), which is highly connected with the communities CM5 (enriched with T cell genes), CM1 (enriched with Monocyte genes) and CM3 (enriched with Neutrophil genes).

A particular subset of neutrophils, neutrophils B-cell helper 2 (NBH2) cells, have low expression of CD15 and CD16 and may help B cells induce immunoglobulin responses in a T cell-independent manner. Compared to conventional neutrophils, NBH2 cells have higher expression of *IL-6*, *IL-10*, *IL-10RB*, *IL-21*, *GRN*, *IDO*, *SOCS1*, *ALDHRA1*, *ARG1*, *TLR8*, *CXCL12*, *CXCL13*, *IL-1B*, *NLRP3*, *NOS2*, *BCL2L1* and *MCL1*, and lower expression of *SLPI*, *TLR9*, *BCL2*, *BAD* and *BAK1* (18). In addition to *IL1B* and *NLRP3*, CM3 contains a lncRNA (IL10RB-DT) that is antisense to the IL10RB gene, which is essential for the IL-10 receptor complex. Two small nucleolar RNA host genes in CM3, SNHG15 and SNHG17, were previously found upregulated in tumor cells and were associated with cell proliferation (19, 20). Their role in CM3 can be related to the proliferation of immune cell types.

Furthermore, monocytes have also been associated with B cell responses and antibody production. In 2014, a research group described that Dengue virus-infected monocytes acquired the CD14+ CD16+ phenotype and were able to elicit the differentiation of B cells into antibody-secreting cells (ASCs) *in vitro* through the secretion of BAFF (TNFSF13B), APRIL (TNFSF13) and TACI (TNFRSF13B) (21). The ligand BAFF was found in CM6 (which is associated with a type I Interferon response), while its 3 possible receptors TACI, BCMA (TNFRSF17) and BAFFR (TNFRSF13C) were found in CM7 (ASC-related genes). One interesting lncRNA in CM1 is the miR-223 host gene. Several papers have shown that miR-223 is highly expressed in activated monocytes/macrophages and that may regulate NLRP3 inflammasome and IL-1B production (22-24). Additionally, NLRP12 also belongs to the CM1 module and its expression is negatively regulated by Blimp-1 (PRDM1), which is needed to

complete B cell differentiation into ASCs (25). Moreover, Blimp-1 is not only expressed during the final steps of the B cell differentiation into ASCs, but also regulates the differentiation of different T cell subsets (enriched in the CM5) (26, 27).

The module CM5 contain genes associated with T cells such as *CARD11*, *CD28*, *CD3E*, *CD3G*, *DLL1*, *DYRK2*, *FYN*, *ICOS*, *ITK*, *MAP4K1*, *NOTCH1*, *PLCG1*, *PRKCA*, *SKAP1*, *STAT4*, and *ZAP70*. In addition to *PRKCQ-AS1* shown in main Fig. 4, there are two other interesting lncRNAs in CM5: LEF1-AS1 and FOXP1-IT1. Beltran et al. (28) have shown that a splicing isoform of LEF1-AS1 was able to attenuate LEF1 mRNA transcription. LEF1 is a key transcription factor that regulates T cell development and differentiation (29). The intronic lncRNA FOXP1-IT1 is able to regulate FOXP1 expression through the IRAK1 pathway (30). Konopacki et al. (31) have shown that FOXP1 has a critical function in Regulatory T cells (Treg) by increasing the DNA binding of FOXP3, and thus enabling efficient IL-2 signaling in these cells. LEF-1 is also a key transcription factor for peripheral Treg homeostasis (32). Finally, Wang et al. (33) demonstrated that LEF1-AS1 may also regulate FOXP1 levels in lung cancer.

Supplementary Figures

Supplementary Figure 1. Reannotation of microarray probes. Intersection between lncRNA probes in platforms used in influenza vaccination studies (a) or YF-17D vaccination studies (b). Barplots located to the left of each figure represent the total number of probes targeting lncRNAs in each platform.

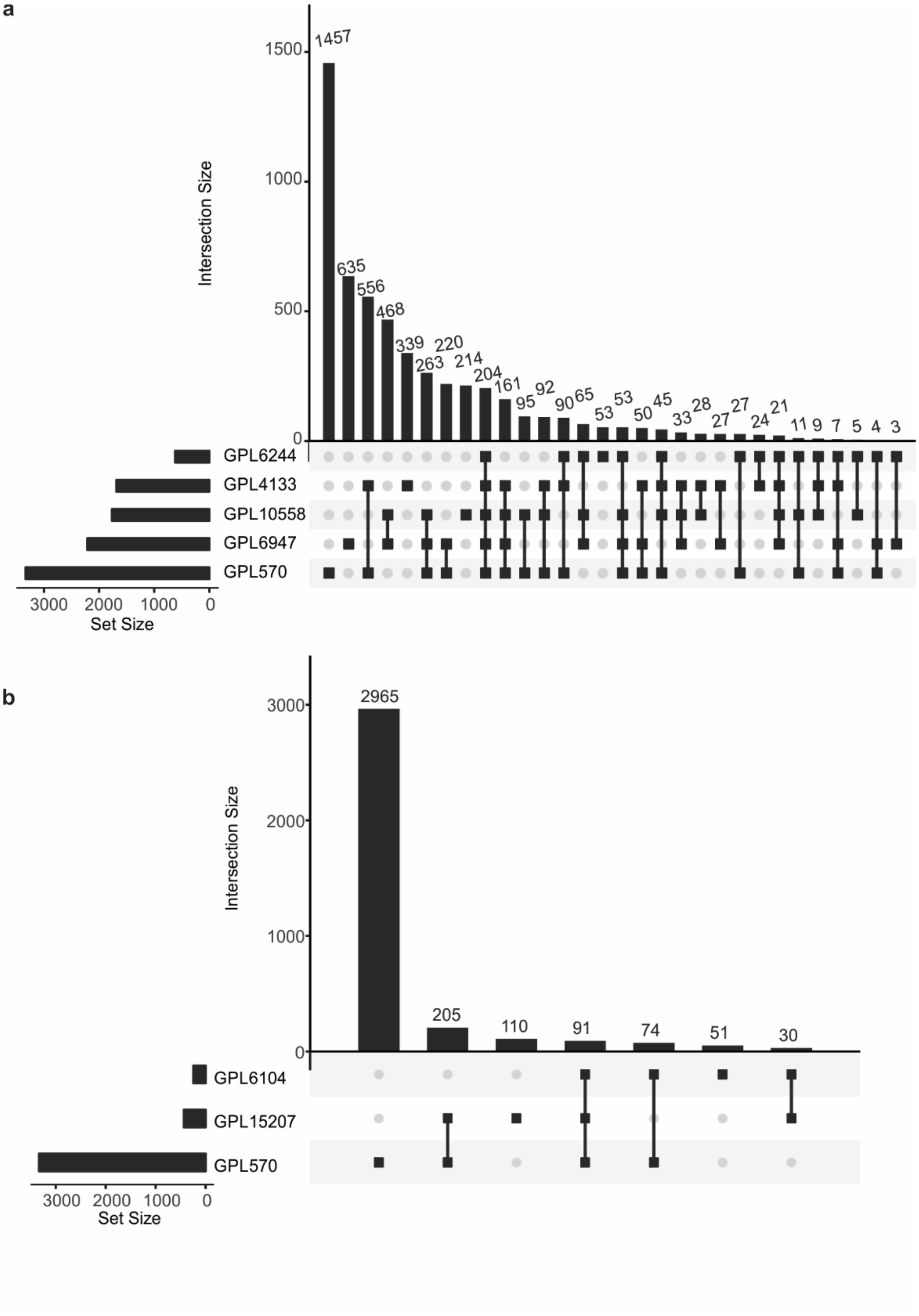

Supplementary Figure 2. Examples of misannotated microarray probes. Genomic regions containing misannotated probes in GPL570 microarray platform.

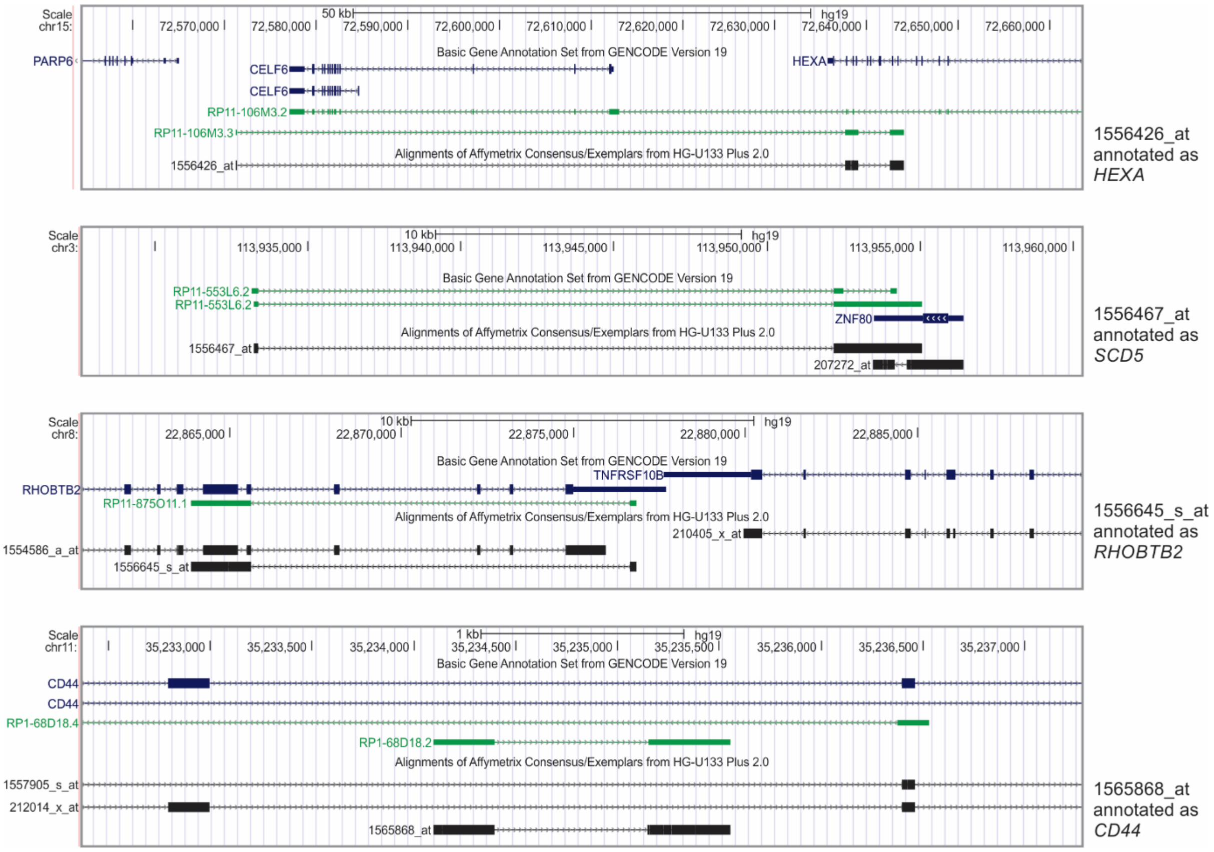

Supplementary figure 3. Vaccination induces changes in expression of lncRNAs. (a) Representatives of lncRNAs whose expression was induced or repressed post-IV. (b) Representatives of lncRNAs whose expression was induced or repressed post-YF17D. Black thick curves and shaded regions represent log2 fold-change summaries relative to baseline and their 95% confidence intervals, respectively.

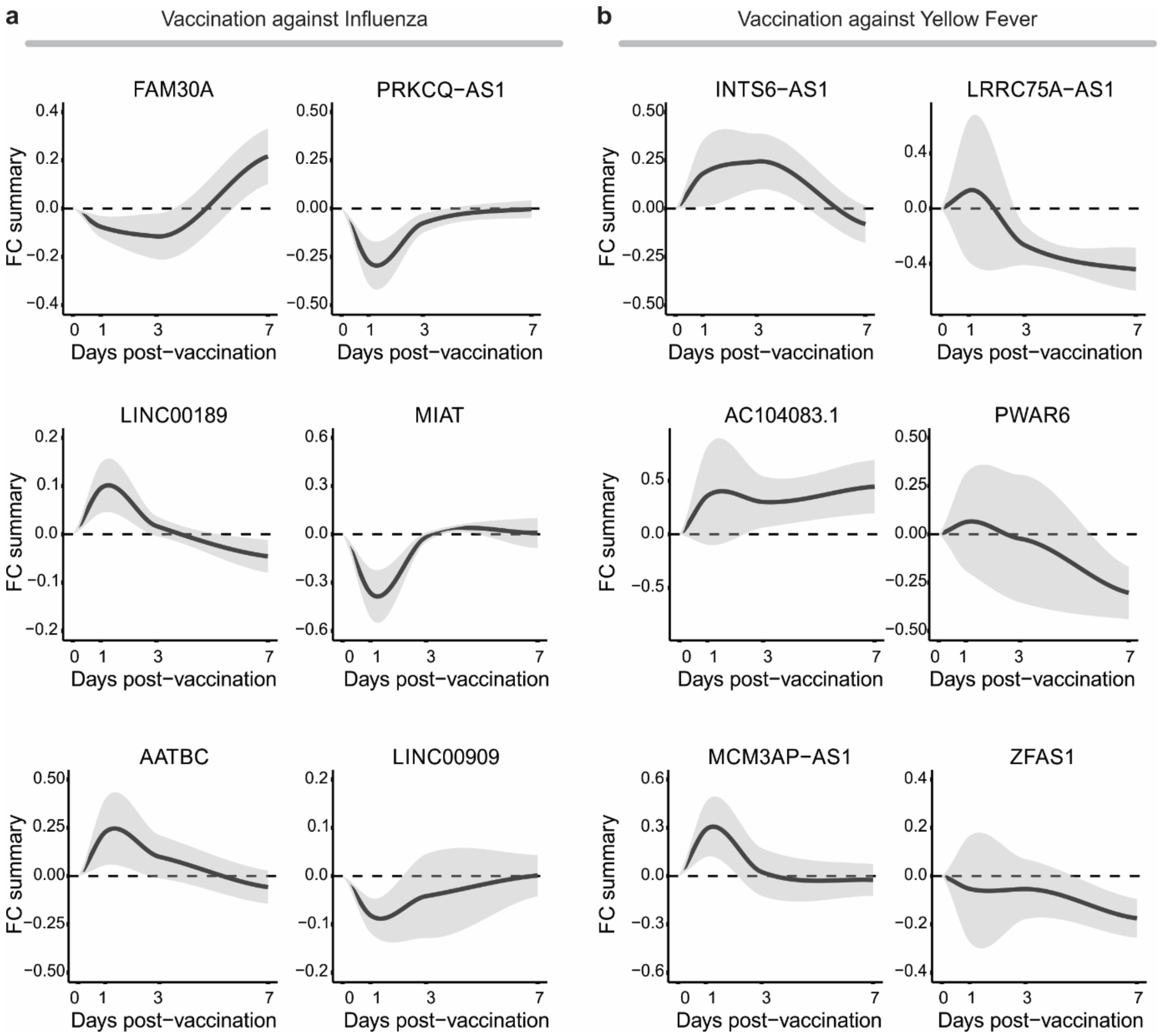

Supplementary figure 4. Gene signatures of influenza vaccination. Heat map of Blood Transcriptional Modules (rows) with changes in activity following vaccination. Gene Set Enrichment Analysis was performed using the means of fold changes between baseline expression values and days 1, 3, 7 and 14 for each cohort (columns) as ranks.

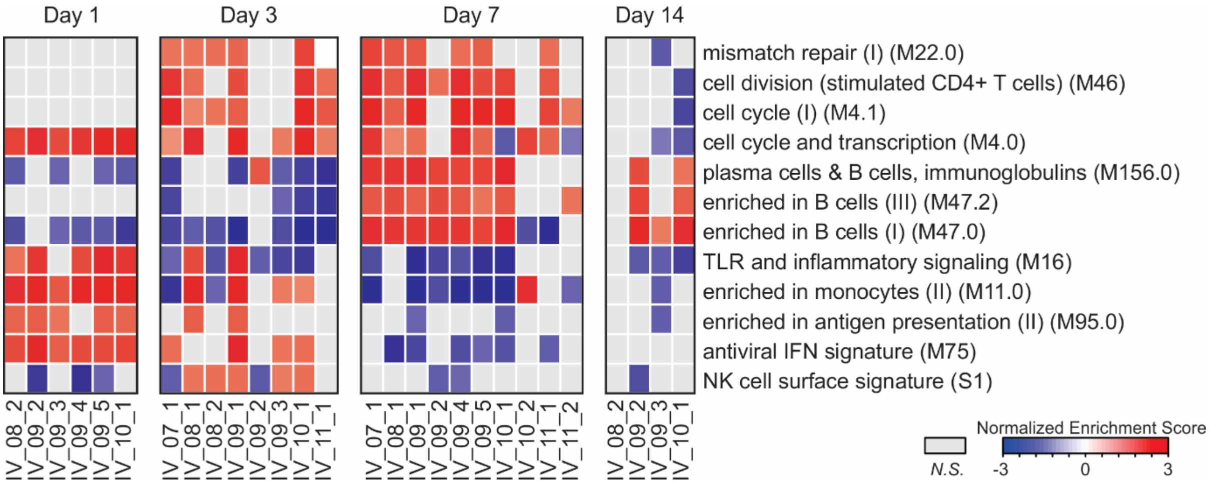

Supplementary Figure 5. Transcriptome analysis of cohorts immunized against yellow fever. Cumulative sum of differentially expressed lncRNAs (a) and genes (b) (y-axis) in one or more datasets (x-axis). Transcripts are considered differentially expressed if limma p-value < 0.05 and they are represented in at least 50% of the datasets. (c) Forest plots of representative lncRNAs with reiterated differential expression. Log2 fold changes with their corresponding 95% confidence interval (x-axis) are plotted for each cohort (y-axis). Red vertical lines and shaded regions represent log_2 fold change summaries and their 95% confidence intervals, respectively. (d) Heatmap depicting gene expression of human immune cells from Blueprint. Log(FPKM) of genes were scaled around zero. Columns represent samples, rows represent genes.

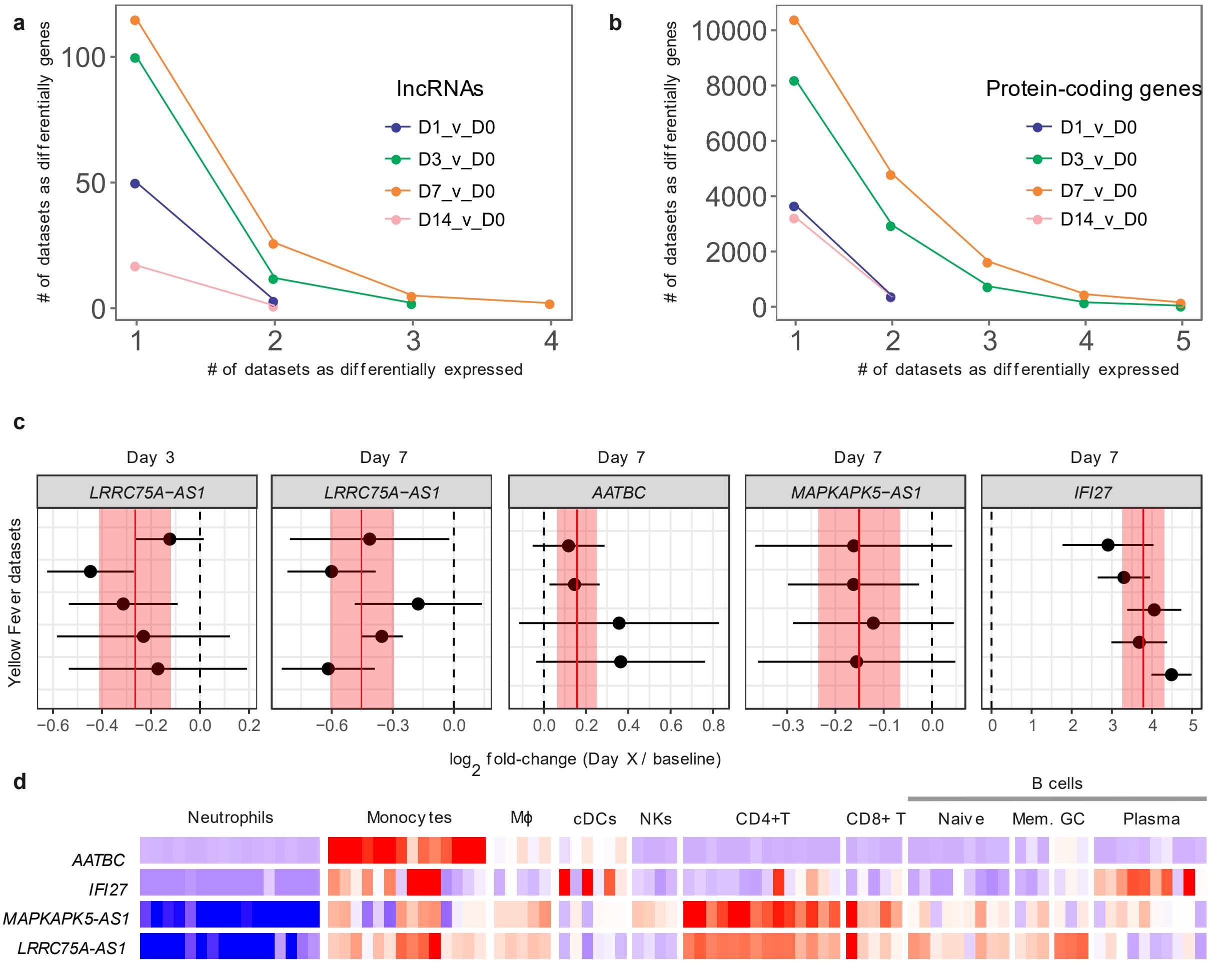

Supplementary Figure 6. YF17D vaccination consensus network. (a) Consensus network was constructed by intersecting CEMiTool results from all YF17D cohorts and prioritizing frequently detected edges. Inference of communities was performed using a spin-glass clustering algorithm. Graph colors are based on community assignment. Each community is represented by a rectangle containing their name and the number of genes and lncRNAs (in parenthesis). (b) Over-Representation Analysis of selected communities using Blood Transcriptional Modules (BTMs). False Discovery Rates (FDR) (x-axis) are plotted for each BTM. (c) Gene Set Enrichment Analysis (GSEA) performed with network communities (rows) and mean fold changes of all vaccinees from each cohort as ranks (columns). Heat map represents Normalized Enrichment Scores (NES) of Communities whose False Discovery Rate (FDR) < 0.05.

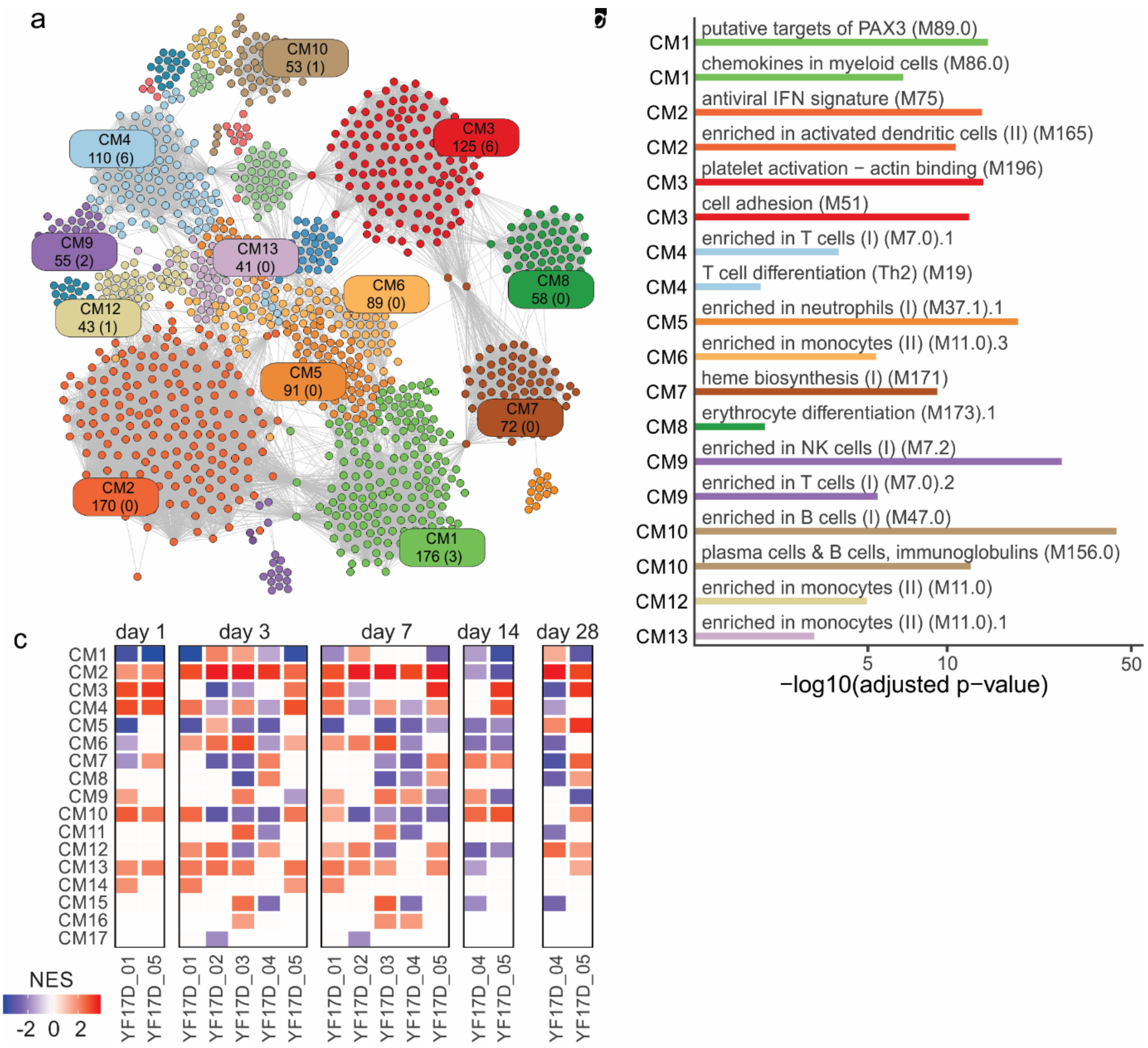

Supplementary Figure 7. Volcano plot of differentially expressed genes at day 2 after vaccination with live attenuated influenza vaccine (LAIV). Dots painted in red represent transcripts annotated as lncRNAs. Dots painted in gray represent transcripts annotated as pertaining to other Ensembl biotype groups. Horizontal dotted line is placed at an FDR threshold of 0.05. Named lncRNAs represent examples of those also found in our meta-analyses from other vaccine cohorts.

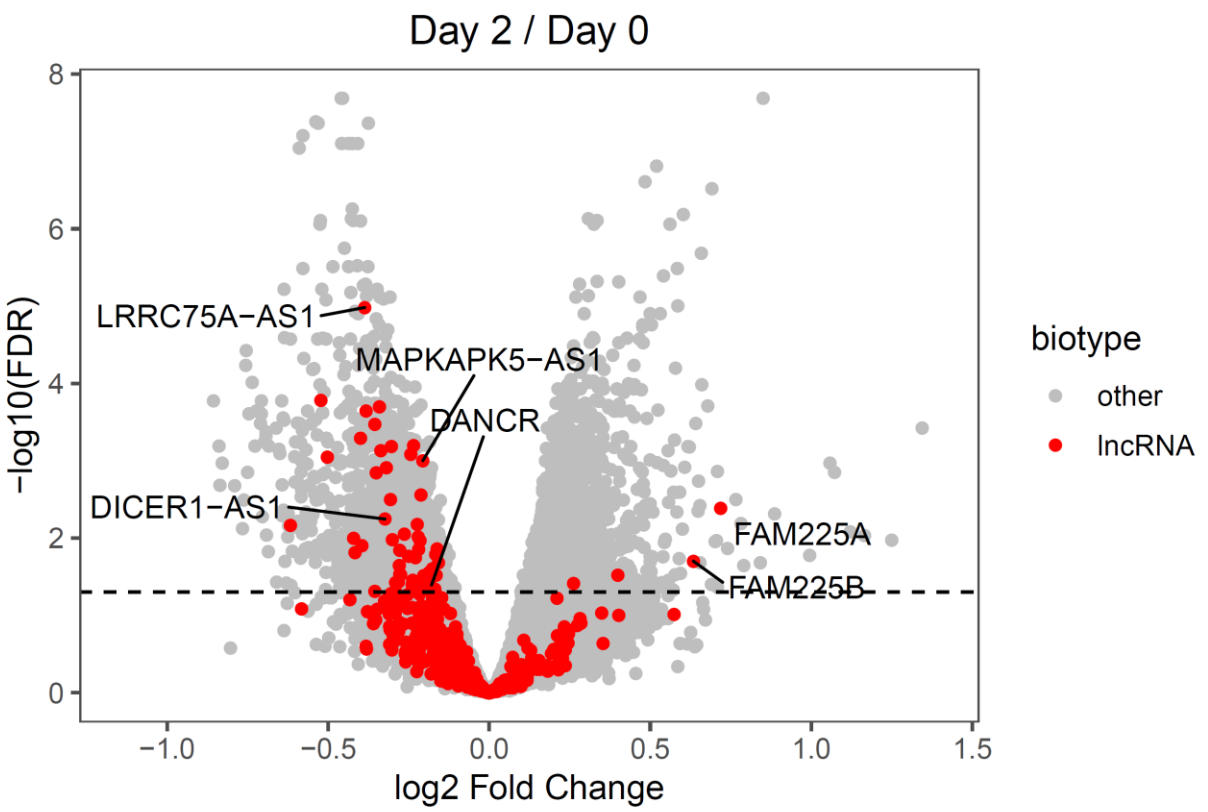

Supplementary Figure 8. Example of VaccineDB results. A query for LRRC75A-AS1 in inactivated influenza vaccine (IV) cohorts was made. Each plot contains aggregated differential expression results of LRRC75A-AS1 as conducted in each day.

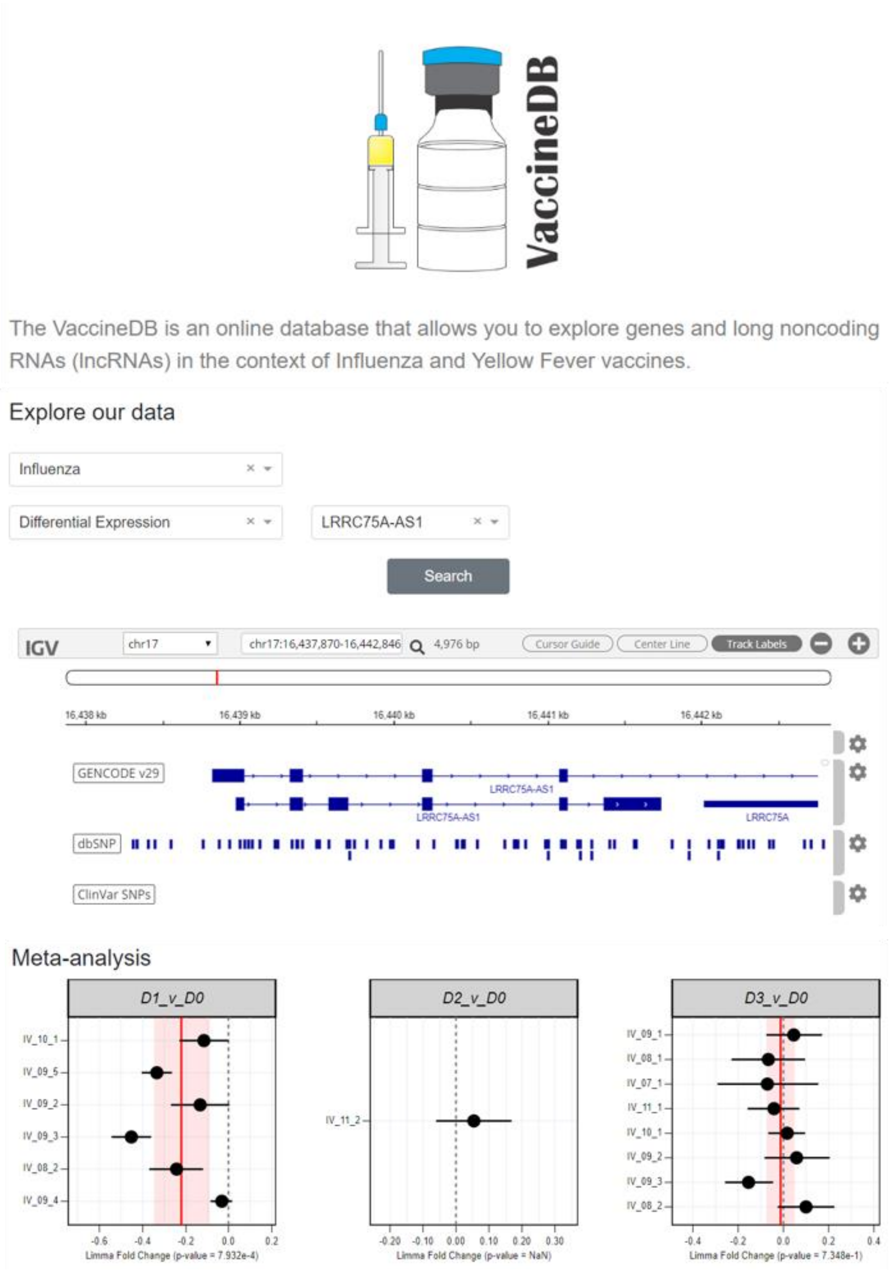

Supplementary Table 1. Vaccination datasets. Abbreviations: Ref – Reference; ID – name of the cohort; Acession – GEO accession ID; AB - Days after vaccination in which antibody titer was measured; Tech. – Techinique used to detect antibody response (MN - Microneutralization assay or HAI - Hemagglutination Assay); YF - Yellow fever; TIV - Trivalent Inactivated Influenza Vaccine; MIV - Monovalent Inactivated Influenza Vaccine; PBMC - Peripheral Blood Mononuclear Cell; WB - Whole Blood; Platform – microarray GEO platform ID.

Ref	ID	Accession	Tissue	AB	Tech.	Vaccine	Platform
(34)	YF17D_01	GSE13486	PBMC	-	-	YF	GPL570
(34)	YF17D_02	GSE13486	PBMC	-	-	YF	GPL570
(35)	YF17D_03	GSE13699	WB	-	-	YF	GPL6104
(35)	YF17D_04	GSE13699	WB	-	-	YF	GPL6104
(36)	YF17D_05	GSE82152	PBMC	-	-	YF	GPL15207
(37)	IV_09_4	GSE47353	PBMC	70	MN	TIV	GPL6244
(38)	IV_08_2	GSE48018	WB	28	HAI	TIV	GPL6947
(38)	IV_09_3	GSE48023	WB	28	HAI	TIV	GPL10558
(39)	IV_09_2	GSE48762	PBMC	-	-	TIV	GPL6947
(40)	IV_10_2	GSE59635	PBMC	-	-	TIV	GPL10558
(40)	IV_11_2	GSE59654	PBMC	-	-	TIV	GPL10558
(41)	IV_09_5	E-MTAB-2313	PBMC	63	HAI	MIV	GPL4133
(42)	IV_10_1	GSE74817/ GSE29619	PBMC	28	HAI	TIV	GPL570
(42)	IV_11_1	GSE74817/ GSE29619	PBMC	28	HAI	TIV	GPL570
(42)	IV_07_1	GSE29619	PBMC	28	HAI	TIV	GPL570
(42)	IV_08_1	GSE29619	PBMC	28	HAI	TIV	GPL570
(42)	IV_09_1	GSE74817/ GSE29619	PBMC	28	HAI	TIV	GPL570

Supplementary Table 2. LncRNAs within communities.

Community	lncRNA
CM1	*AATBC, AC104809.4, DAPK1-IT1, LINC00877, LINC01127, LINC01503, MIR223, RP3-525N10.2*
CM2	*MAPKAPK5-AS1*
CM3	*ALMS1-IT1, CTA-29F11.1, CTD-2033D15.2, CTD-2286N8.2, IL10RB-AS1, LINC00936, MIR222HG, MIR22HG, MIR24-2, RP11-127B20.3, RP11-194N12.2, RP11-373D23.2, RP11-386J22.3, RP11-489E7.4, RP11-670E13.6, RP11-769O8.3, SNHG15, SNHG17*
CM4	*AP001189.4, AP003068.23, C15orf54, RP11-588K22.2, RP11-874J12.4, RP11-879F14.2, RP11-90C4.1, WDR11-AS1*
CM5	*BX322557.10, CHRM3-AS2, CKMT2-AS1, CTD-2555O16.4, FOXP1-IT1, LEF1-AS1, LINC01550, MIR568, PRKCQ-AS1, PWAR6, RP11-138A9.1, RP11-158G18.1, RP11-159N11.4, RP11-161M6.2, RP11-283I3.6, RP11-327P2.7, RP11-395I6.3, RP11-526I2.5, RP11-549J18.1, RP11-664D1.1, SLC25A25-AS1*
CM6	*AC079630.4, RP11-476D10.1*
CM7	*FAM30A, GUSBP11, LINC00926*
CM8	*AC012146.7*
CM9	*A2M-AS1, MIAT, SNHG9*
CM10	*CARD8-AS1, HCP5, ILF3-AS1, LINC00324, RP1-39G22.7, RP11-448G15.3, RP11-53O19.3, RP11-747H7.3, RP11-97C16.1*
CM11	*LRRC75A-AS1, SNHG6*
CM12	*C9orf62, LINC01618, RP11-262I2.2*
CM13	*ITPK1-AS1, LINC00957, LINC01205, RP11-463J7.2, ST8SIA6-AS1*

CM14	*CTB-89H12.4, CTD-2196E14.6, FTX, RP11-104N10.2, SNHG5, SNHG7*
CM15	*LINC01089*

References

1. Kauffmann A, Gentleman R, & Huber W (2009) arrayQualityMetrics--a bioconductor package for quality assessment of microarray data. *Bioinformatics* 25(3):415-416.
2. Ritchie ME*, et al.* (2015) limma powers differential expression analyses for RNA-sequencing and microarray studies. *Nucleic Acids Res* 43(7):e47.
3. Gautier L, Cope L, Bolstad BM, & Irizarry RA (2004) affy - analysis of Affymetrix GeneChip data at the probe level. *Bioinformatics* 20(3):307-315.
4. Kent WJ (2002) BLAT--the BLAST-like alignment tool. *Genome Res* 12(4):656-664.
5. Quinlan AR & Hall IM (2010) BEDTools: a flexible suite of utilities for comparing genomic features. *Bioinformatics* 26(6):841-842.
6. Lawrence M*, et al.* (2013) Software for Computing and Annotating Genomic Ranges. *Plos Comput Biol* 9(8).
7. Russo PST*, et al.* (2018) CEMiTool: a Bioconductor package for performing comprehensive modular co-expression analyses. *BMC Bioinformatics* 19(1):56.
8. Yu GC, Wang LG, Han YY, & He QY (2012) clusterProfiler: an R Package for Comparing Biological Themes Among Gene Clusters. *Omics* 16(5):284-287.
9. Li S*, et al.* (2014) Molecular signatures of antibody responses derived from a systems biology study of five human vaccines. *Nature Immunology* 15(2):195-204.
10. Adams D*, et al.* (2012) BLUEPRINT to decode the epigenetic signature written in blood. *Nat Biotechnol* 30(3):224-226.
11. Albrecht F, List M, Bock C, & Lengauer T (2016) DeepBlue epigenomic data server: programmatic data retrieval and analysis of epigenome region sets. *Nucleic Acids Res* 44(W1):W581-586.
12. Dixit VD*, et al.* (2009) Reduction of T cell-derived ghrelin enhances proinflammatory cytokine expression: implications for age-associated increases in inflammation. *Blood* 113(21):5202-5205.
13. Liu Z, Yan HY, Xia SY, Zhang C, & Xiu YC (2016) Downregulation of long non-coding RNA TRIM52-AS1 functions as a tumor suppressor in renal cell carcinoma. *Mol Med Rep* 13(4):3206-3212.
14. Papatheodorou I*, et al.* (2018) Expression Atlas: gene and protein expression across multiple studies and organisms. *Nucleic Acids Res* 46(D1):D246-D251.
15. Liston A, Papadopoulou AS, Danso-Abeam D, & Dooley J (2012) MicroRNA-29 in the adaptive immune system: setting the threshold. *Cell Mol Life Sci* 69(21):3533-3541.
16. Wang CM*, et al.* (2011) miR-29c targets TNFAIP3, inhibits cell proliferation and induces apoptosis in hepatitis B virus-related hepatocellular carcinoma. *Biochem Bioph Res Co* 411(3):586-592.
17. Chu Y*, et al.* (2011) B cells lacking the tumor suppressor TNFAIP3/A20 display impaired differentiation and hyperactivation and cause inflammation and autoimmunity in aged mice. *Blood* 117(7):2227-2236.
18. Puga I*, et al.* (2011) B cell-helper neutrophils stimulate the diversification and production of immunoglobulin in the marginal zone of the spleen. *Nat Immunol* 13(2):170-180.
19. Kong Q & Qiu M (2018) Long noncoding RNA SNHG15 promotes human breast cancer proliferation, migration and invasion by sponging miR-211-3p. *Biochem Biophys Res Commun* 495(2):1594-1600.
20. Ma ZH*, et al.* (2017) Long non-coding RNA SNHG17 is an unfavourable prognostic factor and promotes cell proliferation by epigenetically silencing P57 in colorectal cancer. *Mol Biosyst* 13(11):2350-2361.
21. Kwissa M*, et al.* (2014) Dengue virus infection induces expansion of a CD14(+)CD16(+) monocyte population that stimulates plasmablast differentiation. *Cell Host Microbe* 16(1):115-127.
22. Haneklaus M*, et al.* (2012) Cutting edge: miR-223 and EBV miR-BART15 regulate the NLRP3 inflammasome and IL-1beta production. *J Immunol* 189(8):3795-3799.

23. Liu YH*, et al.* (2015) miR-223 is upregulated in monocytes from patients with tuberculosis and regulates function of monocyte-derived macrophages. *Molecular Immunology* 67(2):475-481.
24. Neudecker V*, et al.* (2017) Myeloid-derived miR-223 regulates intestinal inflammation via repression of the NLRP3 inflammasome. *Journal of Experimental Medicine* 214(6):1737-1752.
25. Lord CA*, et al.* (2009) Blimp-1/PRDM1 mediates transcriptional suppression of the NLR gene NLRP12/Monarch-1. *J Immunol* 182(5):2948-2958.
26. Martins GA*, et al.* (2006) Transcriptional repressor Blimp-1 regulates T cell homeostasis and function. *Nat Immunol* 7(5):457-465.
27. Johnston RJ*, et al.* (2009) Bcl6 and Blimp-1 are reciprocal and antagonistic regulators of T follicular helper cell differentiation. *Science* 325(5943):1006-1010.
28. Beltran M*, et al.* (2015) Splicing of a non-coding antisense transcript controls LEF1 gene expression. *Nucleic Acids Research* 43(12):5785-5797.
29. Xing S*, et al.* (2019) Tcf1 and Lef1 are required for the immunosuppressive function of regulatory T cells. *J Exp Med* 216(4):847-866.
30. Shi C*, et al.* (2019) Leukocyte integrin signaling regulates FOXP1 gene expression via FOXP1-IT1 long non-coding RNA-mediated IRAK1 pathway. *Bba-Gene Regul Mech* 1862(4):493-508.
31. Konopacki C, Pritykin Y, Rubtsov Y, Leslie CS, & Rudensky AY (2019) Transcription factor Foxp1 regulates Foxp3 chromatin binding and coordinates regulatory T cell function. *Nat Immunol* 20(2):232-242.
32. Fu WX, Yang BH, Yuan XM, & Dong Y (2018) TCF/LEF family transcription factors in peripheral Treg homeostasis. *Journal of Immunology* 200(1).
33. Wang A*, et al.* (2019) LEF1-AS1 contributes to proliferation and invasion through regulating miR-544a/ FOXP1 axis in lung cancer. *Investigational New Drugs*.
34. Querec TD*, et al.* (2009) Systems biology approach predicts immunogenicity of the yellow fever vaccine in humans. *Nat Immunol* 10(1):116-125.
35. Gaucher D*, et al.* (2008) Yellow fever vaccine induces integrated multilineage and polyfunctional immune responses. *J Exp Med* 205(13):3119-3131.
36. Hou J*, et al.* (2017) A Systems Vaccinology Approach Reveals Temporal Transcriptomic Changes of Immune Responses to the Yellow Fever 17D Vaccine. *J Immunol* 199(4):1476-1489.
37. Tsang JS*, et al.* (2014) Global Analyses of Human Immune Variation Reveal Baseline Predictors of Postvaccination Responses. *Cell* 157(2):499-513.
38. Franco LM*, et al.* (2013) Integrative genomic analysis of the human immune response to influenza vaccination. *Elife* 2:e00299.
39. Obermoser G*, et al.* (2013) Systems scale interactive exploration reveals quantitative and qualitative differences in response to influenza and pneumococcal vaccines. *Immunity* 38(4):831-844.
40. Thakar J*, et al.* (2015) Aging-dependent alterations in gene expression and a mitochondrial signature of responsiveness to human influenza vaccination. *Aging-Us* 7(1):38-52.
41. Sobolev O*, et al.* (2016) Adjuvanted influenza-H1N1 vaccination reveals lymphoid signatures of age-dependent early responses and of clinical adverse events (vol 17, pg 204, 2016). *Nature Immunology* 17(6):740-740.
42. Nakaya HI*, et al.* (2015) Systems Analysis of Immunity to Influenza Vaccination across Multiple Years and in Diverse Populations Reveals Shared Molecular Signatures. *Immunity* 43(6):1186-1198.
